# Takotsubo Syndrome is Associated with Mood Disorders and Antidepressants Use, not with Anxiety and Impairment of Quality of Life Due to the Psychiatric Disorder

**DOI:** 10.2174/1745017901814010026

**Published:** 2018-02-27

**Authors:** F. Sancassiani, Mauro G. Carta, Roberta Montisci, Antonio Preti, Sergio Machado, Maria F. Moro, Maria F. Marchetti, Luigi Meloni

**Affiliations:** 1 Department of Medical Sciences and Public Health, University of Cagliari, Cagliari, Italy; 2 Laboratory of Panic and Respiration, Institute of Psychiatry of Federal University of Rio de Janeiro (IPUB/UFRJ), Rio de Janeiro, Brazil; 3 Physical Activity Neuroscience, Physical Activity Sciences Postgraduate Program - Salgado de Oliveira University, Niterói, Brazil

**Keywords:** Tako-Tsubo Syndrome, Mood disorders, Anxiety disorders, Post-traumatic stress disorder, Quality of life, Antidepressants, Psychiatric diagnoses

## Abstract

**Background:**

The aim was to study the association between mood and anxiety disorders and the Tako-Tsubo Syndrome (TTS) and to determine the role of antidepressants and the impairment of quality of life due the comorbid psychiatric disorder.

**Methods:**

Case-control study: 19 consecutive patients (17 female) with TTS compared to 76 controls without TTS, were randomly selected from the database of a nationwide epidemiological study after matching (gender, age and residence) by controls. Psychiatric diagnoses were carried out according to the ICD-10 using semi-structured interview tools (ANTAS-SCID) administered by clinical staff. Quality of Life (Qol) was assessed by means of SF-12.

**Results:**

Only Major Depressive Disorders (MDD) showed higher frequencies in cases with statistical significance difference (p=0.014) as well as at least one Mood Disorder Diagnosis [MDD or BD] (p=0.002). The lifetime prevalence of at least one anxiety disorder with no comorbid mood disorder did not show a higher frequency in cases (p=0.57).

The score at SF-12 in the TTS group was similar to those of controls (p=0.71)In the TTS group, the score at SF-12 in people with one mood or anxiety diagnosis (N=7) was similar to those without mood or anxiety diagnosis (p=0.75). The use of antidepressants was higher in TTS group (15.79% vs 1.31%; p=0.030).

**Conclusion:**

The study shows an association between TTS with depressive disorders and antidepressants use and does not confirm the association with anxiety syndromes. The study suggests the need to investigate the possible interactions between antidepressants use and mood disorders in studies with appropriate design and sample size.

## 1 BACKGROUND

Tako-Tsubo Syndrome (or stress-induced cardiomyopathy) is a reversible clinical entity that mimics acute myocardial infarction. Peculiar features of this syndrome are the lack of evidence of obstructive coronary artery disease on emergent coronary angiography and the transient left ventricular wall motion abnormalities most often involving the apical region that confers to the left ventricle, the typical shape of the pot used for trapping octopus in Japan named as “Tako-Tsubo Syndrome” [1, 2].

Although extensive myocardial dysfunction often resolves rapidly, mortality is comparable with that of patients with coronary acute syndromes [3].

The syndrome usually occurs in postmenopausal women and is typically elicited by emotional or physical stress. An acute increase in cathecolamine surge leading to severe coronary microvascular spasm with myocardial dysfunction has been proposed as the main pathogenetic mechanism and a prior history of mood and anxiety symptoms was found to be associated to this syndrome [4]. The latter has been confirmed by the International Tako-tsubo Registry by showing that lifetime psychiatric affective or anxiety disorders were more frequent in patients with Tako-Tsubo Syndrome than in patients with acute coronary syndrome (55.8% versus with 25.7% of cases) [3].

However, whether the vulnerable substrate for Tako-Tsubo Syndrome is the history of a mood or anxiety disorder in itself, due to the chronic distress, or the antidepressant drugs or, as it seems more likely, both psychiatric illness and treatment, remains unclear. In fact, many antidepressants produce a chronic increase in sympathetic nervous drive in cardiovascular circulation as a side effect of the increase of catecholamines in the central nervous system, that might be amplified acutely by a stressful trigger [5].

An indisputable fact is the several cases of Tako-Tsubo Syndrome reported in the literarture induced as a consequence of convulsant antidepressant therapy in which the elevated levels of catecholamines subsequent to the convulsant antidepressant therapy may be implicated [6-9].

A first important step in understanding the meaning of the association between psychiatric disorders and Tako-Tsubo Syndrome is to see if there is a specific association between particular anxiety or mood disorders. Until now, studies have been conducted using methodologies that only allow broad diagnoses of groups of disorders, such as structured clinical brief interviews [10], questionnaires [11], or rating scales [12], which allow the detection of only anxious or depressive symptoms without a specific diagnosis label.

The aim of this study is to clarify if there is an association between specific mood and anxiety disorders and the Tako-Tsubo Syndrome by using a semi-structured psychiatric interview conducted by clinical staff that allows diagnosis in accordance with the ICD-10 system [13] and a case-control design.

A secondary aim is to determine the impairment of quality of life due to the Tako-Tsubo Syndrome and the role of the concomitant psychiatric disorder, as well the role of concomitant life events.

## 2 METHODS

### 2.1 Design

It was a case control design.

### 2.2 Study Sample

The cases were 19 consecutive patients with Tako-Tsubo Syndrome admitted to the Coronary Care Unit of the University Hospital San Giovanni di Dio in Cagliari, Italy between January 2012 and December 2014. The control group included 76 subjects with no diagnosis of Tako-Tsubo Syndrome randomly selected from the databank of an epidemiological survey on health and mental health in Italy [14-16]. The selection of sex- age- and residence- matched controls from the 3498 subjects was carried out with a randomized block design. A block that included all eligible age-matched (± 1 year), sex-matched and residence-matched (by Italian region) controls in the databank was built for each case. Four individuals per block were drawn for each case, excluding the selected subjects from the remaining blocks.

### 2.3 Psychiatric Diagnosis Interview, Tools, and Psychiatric Assessment

Patients were assessed one month after the acute events. The psychiatric interviews were conducted by means of the “Advanced Neuropsychiatric Tools and Assessment Schedule” (ANTAS) [17]. A semi-structured clinical interview derived in part from the SCID-I non-patient version (SCID-I/NP) [18] was used to assess the presence of psychiatric disorders according to the ICD-10 diagnosis [13].

The ANTAS tool was administered by clinical staff (physicians, psychologists) with adequate training. A previous study [17] pointed out that diagnoses resulting from the ANTAS interview were comparable and reable to those found with SCID.

A questionnaire on psychotropic drug consumption, prescription circumstances and health services utilization was also adminstered in cases and controls [17].

The perceived quality of life was measured by means of the Short Form Health Survey (SF-12) [19] in the Italian version [20]. The SF-12 components are the seven dimensions: activity, limitations due to health conditions, emotional state, physical pain, perception of general health, vitality, social relationship and psychosocial disability. The tool measures the month prior to evaluation and higher scores correspond to a better quality of life.

### 2.4 Diagnosis of Tako-Tsubo Syndrome

The Mayo Clinic criteria were followed in diagnosing Tako-Tsubo Syndrome [21, 22], including:

1. Transient regional left ventricular systolic dysfunction;
2. Absence of obstructive coronary disease or acute plaque rupture (on angiography);
3. New mild or moderate ECG abnormalities (such as ST elevation or T inversion) or elevation in cardiac troponin;
4. Exclusion of pheochromocytoma or myocarditis.

### 2.5 Screening Controls for Tako-Tsubo Syndrome

During the interviews of the controls, each was asked about general wellbeing, consultation with physicians and diagnosis of illness, and medical tests they underwent routinely (e.g., work/education periodical controls or driver’s license or hunter’s license eligibility tests). Diagnosis of physical illness was reported on a structured form. People with acute or chronic myocardiopathy were excluded.

## 3 DATA ANALYSIS

Lifetime prevalence of ICD-10 [13], Major Depressive Disorder (MDD), Bipolar Disorder (BD) (including Bipolar I and Bipolar II), Post-Traumatic Stress Disorder (PTSD), Panic Disorder (PD) and Generalized Anxiety Disorder (GAD) were calculated in cases and controls. The odds ratio for each ICD-10 diagnosis, as well as for all mood disorders, for all anxiety disorders without comorbidity with mood disorders and for all mood or anxiety disorders (dependent variables) were calculated using the control group as a “pivot” with univariate analysis (owing to the matching method, the groups were balanced by age and gender). The analysis of variance (ANOVA one-way) was used for measuring the parametric variables; the χ^2^ test was used for nonparametric variables. The odds ratio and 95% confidence intervals (OR 95% CI) were calculated with Miettinen’s simplified method.

## 4 RESULTS

The study samples were represented by 19 cases (17 female) with a diagnosis of Tako-Tsubo Syndrome and by a control group of 76 subjects (68 females) without a diagnosis of Tako-Tsubo Syndrome; the matching method allowed controls to be perfectly homogeneous with cases by age and gender. The characteristics of cases and controls are reported in Table **1**.

Table **2** shows the lifetime prevalence and the measure of association of psychiatric disorders (MDD, BD, PTSD, PD and GAD) as well as all people with at least one mood disorder and all people with at least one anxiety disorder without comorbidity with mood disorders; all people with at least one mood or anxiety disorder in cases and controls. All psychiatric disorders evaluated in this study showed a higher prevalence in cases, but only in MDD, the difference reached statistical significance (26.3%)% *vs* 6.57%; χ^2^= 6.27, p=0.014) as well as the association of people with at least one mood disorder diagnosis and Tako-Tsubo Syndrome (31.5% *vs* 6.57%; χ^2^= 9.27, p=0.002). On the contrary, the lifetime prevalence of people with at least one anxiety disorder without mood disorder showed a low frequency in patients with Tako-Tsubo Syndrome. The difference versus controls did not reach statistical significance (10.52% vs 3.95%; χ^2^ with Yates correction 0.33, p=0.57).

The SF-12 mean score±sd in the Tako-tsubo group was 34.76±6.92, *versus* 35.35±6.23 in the control group (ANOVA 1 way: df 1,93,94; F=0.4; p=0.71); the SF-12 mean score±sd in people with one mood or anxiety diagnosis in the Tako-tsubo group (N=7) was 34.00±2.92, *versus* 35.17±9.25 in people of the Tako-tsubo group without anxiety or mood diagnosis (ANOVA 1 way: df 1,17,10; F=0.104; p=0.75). In 17 out of 19 (84.47%) patients with Tako-Tsubo Syndrome, at least one relevant life event (such as death or severe illness of a relative, job loss, serious bickering in family or with friends) was found one month prior to hospitalization. The two cases without relevant life events showed no association with psychiatric disorders, and the frequency of prior life events in people with Tako-tsubo and psychiatric disorders compared to those with Tako-tsubo but without them (Fisher exact test = 0.508) showed no statistical significance.

The use of antidepressants in the month prior the interview, was found in 3 (15.79%) out of patients with Tako-Tsubo Syndrome and in 1 (1.31%) out of subjects in the control group (χ^2^ with Yates correcrion =4.71; p=0.030; OR=14.06; CI95% 1.17-376.14). Out of 3 cases using antidepressants in Tako-tsubo group, 1 was diagnosed of MDD, 1 of BD and 1 had no diagnosis of mood disorders at ANTAS-SCID; the control using antidepressants diagnosed MD. After direct standardization considering the freqeuency of mood disorder in the control group, the association with the use of antidepressants and Tako-Tsubo Syndrome became weak, and it was found at the limit of the statistical significance (9.3% *vs* 1.3%; χ^2^=0.064).

## 5 DISCUSSION

Our work has confirmed the association of the Tako-Tsubo Syndrome with mood disorders, and with depressive disorders in particular. Our data do not appear to confirm the association between Tako-tsubo and anxious syndromes previously reported in literature [10, 12, 23].

The study highlighted an association between Tako-Tsubo Syndrome and the use of antidepressants, but the sample is too small to perform a multivariate analysis to study the interaction of the two factors. The standardization of the case sample based on the frequency of mood disorders in the controls reduces the strength of the association between Tako-Tsubo Syndrome and the use of antidepressant drugs (in this way irrespective of the presence of mood disorders) at the limits of the statistical significance.

The divergence in the association with Tako-tsubo e Anxiety Disorders shown in literature data may be because this is the first study to have used diagnoses according to international criteria based on semi-structured clinical interviews conducted by the clinical staff [17, 23]. In our survey, we separately analyzed the patients with anxiety disorders from those with comorbid anxiety-depression in order to understand if anxiety alone may be a determinant or if comorbidity with mood disorder could be a confounding factor.

Some previous studies used an anxiety disorder diagnosis in accordance with international classification systems [24] such as DSM-IV TR [18]. However, the diagnosis was based on clinical records and therefore, not verified through semi-structured interviews, which is recognized as the method most capable to producing reliable diagnoses in psychiatry. Other surveys adopted screening by brief structured clinical interviews [10], questionnaires [11], or rating scales [12] but this is the first study to the best of our knowledge that used a more complex methodology.

Anxiety and depression are often associated, especially in cardio-circulatory disorders [25-28]. However, in the broad spectrum of mood and anxiety disorders, there are different disorders requiring even different treatments. Although many antidepressants are also indicated in anxiety disorders, they are rarely used in anxiety disorders not associated with depression in Italy [29].

If one attempts to gain a better understanding of whether the association of Tako-Tsubo Syndrome with depressive disorders is at least partly due to the use of antidepressants, adequate diagnoses should be used and the anxiety and depression disorders should be clearly distinguished.

On the other hand, the results may be affected by the fact that a study with a small sample size does not have the power to show differences in the association of Tako-tsubo with anxiety disorders that would have emerged in a larger sample. In any case, it must be emphasized that anxiety disorders have high rates of prevalence in the community: for example, a 12-month prevalence of 1.9-5.1% only for Generalized Anxiety Disorders (GAD) [30]. With similar or even lower frequencies in the community, depressive disorders were found to be associated with Tako-Tsubo Syndrome in our sample. It has often been found that Tako-Tsubo Syndrome can be a consequence of stress and that the underlying element of the association may be due to the high stress associated with depressive anxiety disorders [10].

The data of our study neither reinforce nor contradict this hypothesis, but are based on theories that depressive lifetime disorders may lower the vulnerability to stress and to the stress linked to life events [31]. In our sample, we found high percentages of both depressive disorders and life events in people with Tako-Tsubo Syndrome; these are the elements that may suggest a role of depressive disorders per se, independently of the treatment as a possible causal factor. On the other hand, the cases in our observations showed no impairment of the quality of life of those with Tako-Tsubo Syndrome only or, above all, of those with associated mood disorder, so that one may suspect that the effect on stress of the mood disorders alone may not be sufficient to support that the cause of the association is the depressive disorders alone.

## 6 LIMITS

A limitation of this survey is the fact that, while the cases received a diagnosis of Tako-Tsubo Syndrome in a clinical setting according to the standard criteria, in the control sample, the diagnoses were based only on medical history and previous investigations. Thus, a number of people who screened negative for Tako-Tsubo Syndrome may be undiagnosed. But this limit does not detract from the relevance of the association with mood disorders, because, in the light of possible false positives in the control group, it will only decrease the measure of association between cases and controls. Also given the low frequency of the Tako-Tsubo Syndrome, the chance of a case between the controls is really low.

## CONCLUSION

Our work, first carried out with psychiatric semi-structured interviews administered by clinicians, has confirmed the association of the Tako-Tsubo Syndrome with mood disorders and with depressive disorders in particular.

The results do not appear to confirm the association between Tako-Tsubo Syndrome and anxious syndromes.

Data on the use of antidepressants suggests an effect of these drugs regardless of the effect of depression but the results are not conclusive. The study suggests the need for studies with adeguate sample size and methods to better define if there is an independent role of the two factors (depressive disorders and antidepressants use) and/or a possible interaction.

## Figures and Tables

**Table 1:** Demographic characteristics of cases and controls.

Characteristics	**Cases**	**Controls**
**Sample size**	19	76
**Gender (Female)**	17	68
**Age (mean ± sd)**	63.26 ± 9.21	63.26 ± 9.03

**Table 2:** Lifetime prevalence of ICD-10 psychiatric disorders in cases and controls.

Psychiatric Disorders	**Cases**	**Controls**	**χ^2^**	**p**	**OR**	**CI 95%**
	**(N=19)**	**(N=76)**				
*Major Depressive* *Disorder*	5 (26.3%)	5 (6.57%)	6.27	0.014	4.92	1.05 – 23.52
*Bipolar* *Disorder*	1 (5.26%)	0 (0%)	0.57 *	P=0.45	Not Calc	-----
*Post-Traumatic Stress Disorder*	1 (5.26%)	2 (2.63%)	0.02*	0.98	2.13	0.08-35.95
*Generalized Anxiety Disorder*	2 (10.53%)	2 (2.63%)	0.80*	0.37	4.35	0.40-47.74
*Panic Disorder*	1 (5.26%%)	3(3.94%)	0.01*	0.99	1.35	0.05- 16.19
*Any Mood* *Disorder*	6 (31.5%)	5 (6.58%)	9.28	0.002	6.55	1.47-30.09
*Any Anxiety Disorder* *(without Mood Disorder)*	1 (5.26%)	3 (3.94%)	0.01*	0.99	1.35	0.05-16.19
*Any Mood or Anxiety* *Disorder*	7 (36.84%)	8(10.52%)	9.64	0.002	5.95	1.52-23.19
*Use of Antidepressants in the last month*	3(15.79%)	1 (1.31%)	4.71*	0.030	14.06	1.17-376.14
*Use of Antidepressants in the last month, standardized by* *Depressed in controls*	1,77(9.31%)	1 (1.31%)	3.43*	0.067	7.70	0.45-238.21

*Yates correction
